# New Technologies as Promising Tools for Assessing Facial Emotion Expressions Impairments in ASD: A Systematic Review

**DOI:** 10.3389/fpsyt.2021.634756

**Published:** 2021-05-05

**Authors:** Kellen Briot, Adrien Pizano, Manuel Bouvard, Anouck Amestoy

**Affiliations:** ^1^Medical Sciences Department, University of Bordeaux, Bordeaux, France; ^2^Pôle Universitaire de Psychiatrie de l'Enfant et de l'Adolescent, Centre Hospitalier Charles-Perrens, Bordeaux, France; ^3^Aquitaine Institute for Cognitive and Integrative Neuroscience (INCIA), UMR 5287, CNRS, Bordeaux, France

**Keywords:** autism (ASD), facial expression, emotion, new technologies, automatic facial expression analysis

## Abstract

The ability to recognize and express emotions from facial expressions are essential for successful social interactions. Facial Emotion Recognition (FER) and Facial Emotion Expressions (FEEs), both of which seem to be impaired in Autism Spectrum Disorders (ASD) and contribute to socio-communicative difficulties, participate in the diagnostic criteria for ASD. Only a few studies have focused on FEEs processing and the rare behavioral studies of FEEs in ASD have yielded mixed results. Here, we review studies comparing the production of FEEs between participants with ASD and non-ASD control subjects, with a particular focus on the use of automatic facial expression analysis software. A systematic literature search in accordance with the PRISMA statement identified 20 reports published up to August 2020 concerning the use of new technologies to evaluate both spontaneous and voluntary FEEs in participants with ASD. Overall, the results highlight the importance of considering socio-demographic factors and psychiatric co-morbidities which may explain the previous inconsistent findings, particularly regarding quantitative data on spontaneous facial expressions. There is also reported evidence for an inadequacy of FEEs in individuals with ASD in relation to expected emotion, with a lower quality and coordination of facial muscular movements. Spatial and kinematic approaches to characterizing the synchrony, symmetry and complexity of facial muscle movements thus offer clues to identifying and exploring promising new diagnostic targets. These findings have allowed hypothesizing that there may be mismatches between mental representations and the production of FEEs themselves in ASD. Such considerations are in line with the Facial Feedback Hypothesis deficit in ASD as part of the Broken Mirror Theory, with the results suggesting impairments of neural sensory-motor systems involved in processing emotional information and ensuring embodied representations of emotions, which are the basis of human empathy. In conclusion, new technologies are promising tools for evaluating the production of FEEs in individuals with ASD, and controlled studies involving larger samples of patients and where possible confounding factors are considered, should be conducted in order to better understand and counter the difficulties in global emotional processing in ASD.

## Introduction

Non-verbal information extracted from facial expressions are crucial components of social functioning and impairments to such behavior have a strong impact on social interactions, since facial expressions provide a window to internal emotional state and are key to successful communication and social inclusion. In the general population, it is largely recognized that reciprocity and synchrony in facial expression production has an important social function and that atypical facial expressions are correlated with weaker social skills (1). Autism Spectrum Disorders (ASD) are neurodevelopmental disorders characterized by difficulties with social communication and interaction associated with stereotyped and repetitive behaviors, restricted interest and sensory abnormalities, according to the DSM-V (2). In ASD, the deficit observed in social skills represents one of the major and persistent characteristics of ASD's core symptoms and includes impairments in social and/or emotional reciprocity and in the use and understanding of verbal and non-verbal communication. These in turn lead to difficulties in modulating and maintaining adapted social behaviors, even in individuals with High Functioning Autism (HFA) who often struggle in social settings because of difficulty in interpreting and producing facial expressions. Therefore, Facial Emotion Recognition (FER) has been a topic of interest in autism research for more than three decades, although studies have generally documented mixed results that have failed to provide a consensus on these impairments in both children and adults with ASD (3). In addition to the deficit in FER (4), a deficit in the production of FEEs, which is part of non-verbal communication, is also a frequently reported symptom in ASD. FEEs are often perceived as awkward or atypical and are judged as clinically relevant measures in the Autism Diagnostic Observation Schedule - Second Edition (ADOS-2) (5) or the Autism Diagnostic Interview - Revised (ADI-R) (6), which are the most commonly used tools in the ASD diagnosis process.

The production of facial expressions has been proposed to be linked to a specific neural network corresponding to the Mirror Neuron System (MNS) that in humans includes the pre-motor cortex, the inferior parietal and frontal lobes and their interactions with the limbic system, the insula and the anterior cingulate cortex. In non-ASD individuals, this network is activated during the execution of a motor action as well as during the observation of a motor action performed by others (7). Evidence for MNS impairments in ASD has been taken to support the hypothesis that the MNS plays a role in higher order socio-cognitive functions that help us to understand another person's perspective and inner states, such as action understanding, theory of mind, emotion simulation and empathy (7, 8). Accordingly, it has been suggested that deficits in the production of FEEs may be related to a MNS dysfunction, particularly in ASD, in line with the general MNS dysfunction theory: the broken mirror theory (BMT) in ASD (9). However, despite the appealing simplicity of the BMT, evidence supporting the hypothesis is mixed, with a recent review distinguishing three variants of the BMT that highlight contradictory results in imitation, simulation and emotion recognition tasks (10). A MNS dysfunction limited to specific stimuli could better explain the abnormal modulation of social cues (10).

Related to the embodiment theory, the facial feedback hypothesis suggests that in non-ASD individuals, the experience of emotions is affected by feedback from facial muscle activation (11). Stel et al. demonstrated that automatic or voluntary facial expressions, modulated by holding a pen between the teeth, influenced corresponding emotions compared to non-pen holding in controls, while adolescents with ASD remained emotionally unaffected. The authors concluded that the facial feedback mechanism worked differently for individuals with ASD (12). Moreover, previous results reported that disrupting sensory feedback from the facial region of the somatosensory cortex using Transcranial Magnetic Stimulation (TMS) impaired the discrimination of facial expressions, but not the recognition of facial identity (13). Together, these studies suggest that perception and motor production may be linked through a common use of the motor system, in line with the perception-action coupling system theory. Similarly, it is recognized that people with ASD may also present atypical mental representations of their emotional experiences (14). A deficit in the perception-action coupling could, in ASD, partly explain both impairments: in sensory mental representations of emotion and motor programming of facial expression. The sensory-motor process plays an important role in the mentalization of one's internal states and intentions.

When engaged in a social interaction, typically developing individuals fail to interpret the facial expression of people with ASD (15), with difficulties to identify and discriminate the emotion expressed (e.g., not being able to discern a sad face from an angry face) (16). Furthermore, unfamiliar interlocutors judge individuals with ASD less favorably on the basis of their non-verbal cues, and are therefore less likely to engage in a social relationship because of an impression of “weirdness” (17). As a result, people with ASD are more likely to be judged more unfavorably by non-ASD peers, they develop less friendships and are more vulnerable to bullying, especially at school (18).

However, results concerning FEEs in ASD remain highly variable among traditional studies, without the use of new technology, partly due to both task and participant characteristic factors such as age and intellectual functioning (19). In addition, several clinical dimensional traits impact the production of FEEs, such as alexithymia [which involves difficulties in recognizing and distinguishing between different emotions and body sensations, difficulties in expressing emotions, lack of imagination or fantasy life, and thoughts focused on external rather than internal experiences (20)] (21), or depressive symptoms (22). Moreover, facial expressions have been studied through the employment of different tools. The Facial Action Coding System (FACS) (23), which was developed by Ekman in 1976, is the most frequently used tool in traditional research on FEEs. The FACS is a human rated system for objectively scoring facial expressions in terms of elemental movements, called action units (AUs), which correspond approximately to individual facial muscle movements. FACS provides a comprehensive description of facial expressions, imparting a greater specificity and diversity than emotion categories. Facial electromyography (EMG) is another commonly used method that is proposed to measure selective facial muscle contractions during facial expression.

These conventional techniques have several disadvantages in addition to a lack of reliability. The FACS has been widely used for research on emotions, however it involves manual human coding and is therefore less reliable and objective. Moreover, it is likely to be difficult to apply to dynamic facial expressions. Particularly, human coding involves time and training requirements and above all cannot be used in real time or in an ecological social environment. EMG recordings are mainly limited to the two groups of muscles responsible for frowning (*corrugator supercilii*) and smiling (*zygomaticus major*) and does not allow detailed analysis of all regions of the human face. EMG analysis is also invasive and the required use of markers on the face can interfere with the natural facial expression of emotion. However, recent advances in automated facial expression recognition technology has opened new possibilities for the objective measurement of facial expressions, with both qualitative and quantitative advantages.

New technologies can be defined as the use of mechanical or electromechanical procedures that increase productivity and reduce or eliminate manual operations or operations performed by older technologies (24). They include mobile phones, video recording equipment, robotics, computers etc. The interest for the analysis of FEEs is multiple (25). First, the advent of automated facial expression analysis software and resultant reductions in analysis time allows researchers to obtain larger samples of individuals with ASD and collect in-depth information through real-time analysis of multiple and complex relevant characteristics. Second, the data obtained are recorded automatically, which makes distinguishing differences between participant groups more objective than those obtained with human observers and raters. Then, compared to studies based on human coders, it has been reported that automated FEE analysis were able to achieve high test–retest reliability across healthy, non-ASD participants (26).

The present systematic review therefore aims to report recent research using new technology based on automated facial expression recognition systems that allow a more objective and detailed characterization of quantitative and qualitative deficits of FEEs in individuals with ASD.

## Materials and Methods

This review focuses on the use of automated facial recognition technology to analyze facial expression in ASD subjects. We have not included either studies that examined only facial emotional recognition (FER) or other non-affective facial processing, nor studies based on observer rating of facial expressions or EMG monitoring (so called “traditional studies or literature”), but rather have focused uniquely on the automated evaluation of emotional facial expressions based on new technologies. This review is in accordance with the Preferred Reporting Items for Systematic Reviews and Meta-analyses (PRISMA) statement (27).

The data bases used were: PubMed database for medical sciences, the IEEE Xplore database for new technologies, computer science and engineering, and the Web of Science database using a combination of the following MESH terms or keywords: (asd OR Autism OR Asperger) AND (facial OR face) AND (emotion OR emotion expression OR emotion recognition OR expression OR production) AND (software OR technology OR comput^*^ OR automat^*^ OR technology-based intervention). A manual search was also performed by checking the list of References of the studies included in this review. Articles reporting experimental and clinical studies, literature reviews or meta-analyses were considered.

To be included in the systematic review, studies also had to meet the following criteria:

- the diagnosis of ASD must have been made formally by a specialist using the diagnostic criteria of the DSM-IV, DSM-IV-TR, DSM-V, or the International Classification of Diseases 10th Edition ICD-10 or ICD-11 to allow maximum generalizability to current practice,
- specification of the age or age category (child/adult) of the participants included.,
- specification of the type of stimuli used and the method of facial expression analysis,
- comparative studies that included a control group of participants without ASD
- studies written in English and published in scientific journals until August 2020.

In a first instance, 1,749 relevant results were found. After exclusion of duplicates (*n* = 660), the titles and abstracts of the remaining 1,089 articles were analyzed according to the above criteria.

**Figure 1:**
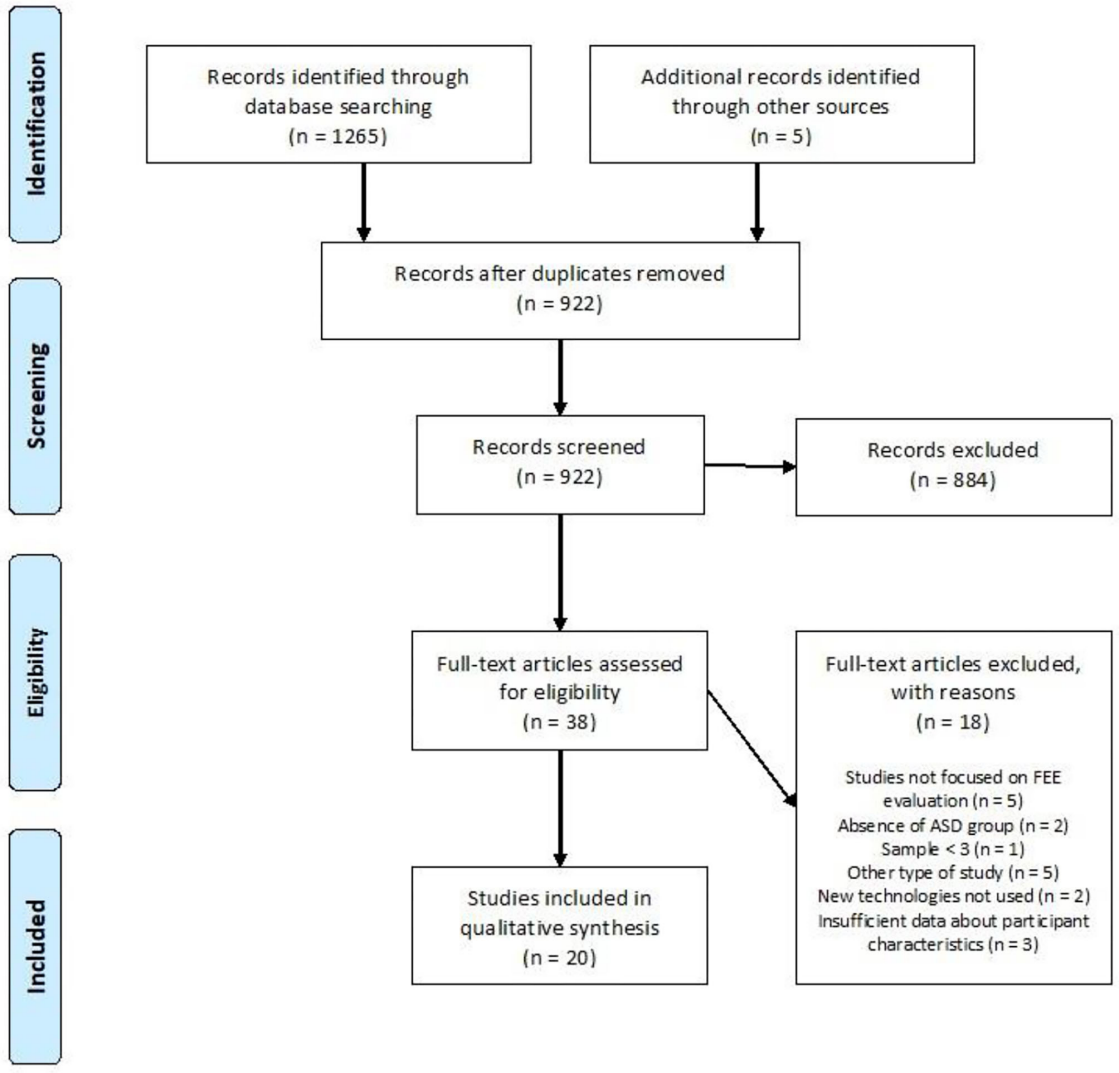
Flow diagram.

Initially, two of this review's authors (KB, AA) evaluated the title and abstract of the selected articles to determine whether they met the inclusion and exclusion criteria. One thousand thirty-six articles were subsequently excluded and 53 were read in full. The studies selected from this first analysis were independently examined by the two authors, who read the full text. A further 33 articles were excluded because (1) they did not consider facial expression but recognition of emotion (*n* = 15), (2) they did not include a specific group of individuals without ASD (*n* = 5), (3) they included a number < 3 of subjects with ASD or were a case study (*n* = 1), (4) they were not an original study or a literature review or meta-analysis (*n* = 5), (5) they did not involve new technologies (*n* = 4), (6) the methodological description or description of the study population was too limited to ensure comparability with other studies (*n* = 3). Ultimately, 20 articles were included in our literature review. The associated flow chart is presented in Figure 1.

**Figure 2:**
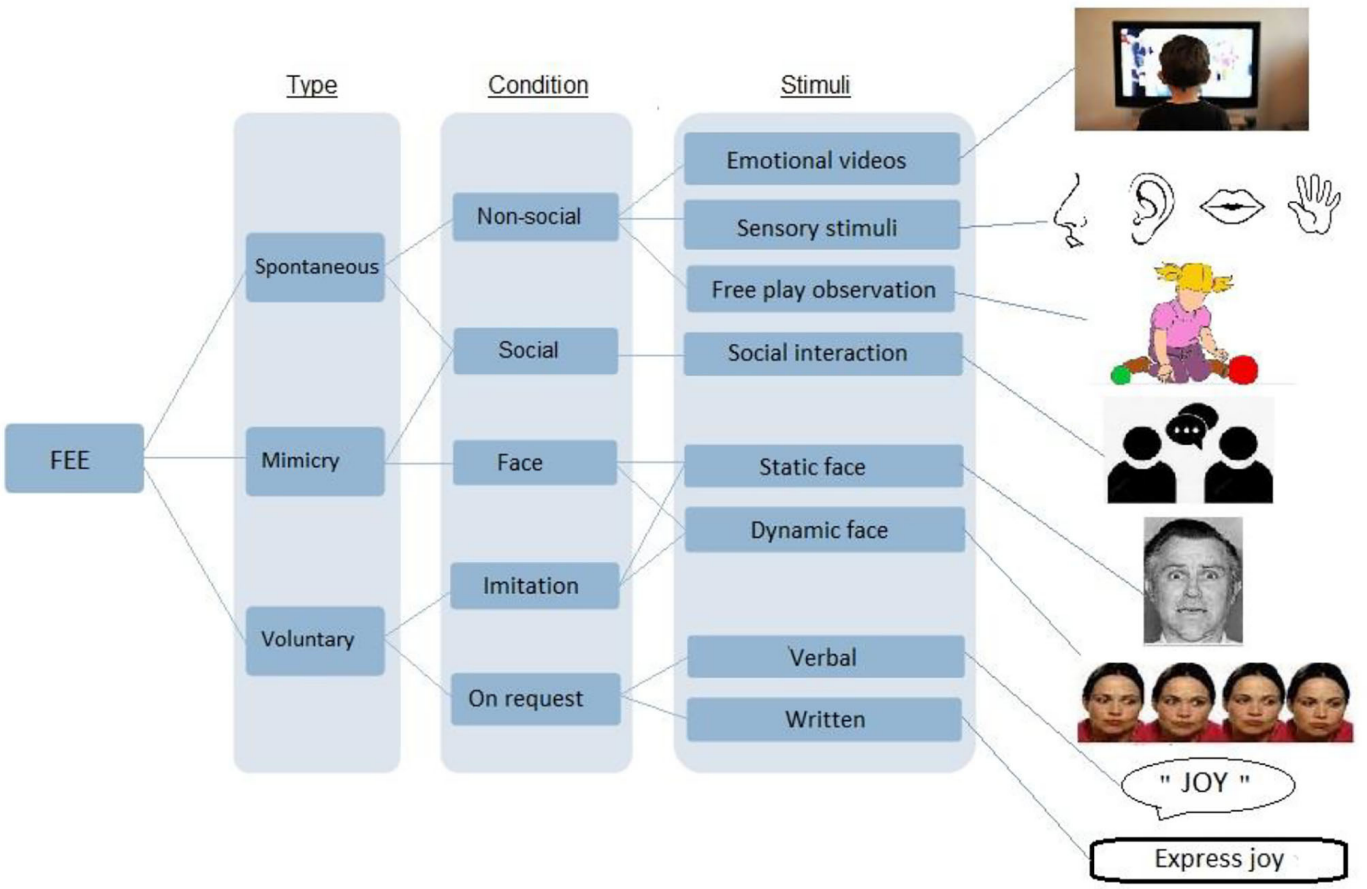
Description of the different types of stimuli used in facial expression analysis study methodologies.

Each variable related to participant parameters, the methods used and the study results as presented in Table 1 for clinical studies and in Table 2 for reviews and meta-analysis. Participant parameters included were group size, age, gender, intellectual functioning, and method of ASD clinical assessment method. Methods of facial expression analysis included the type of stimulus provided (see Figure 2 for a description), the type of measurement, and the type of expression (spontaneous or voluntary). Additional scales used were identified and a summary of the results was made. The quality of each study was evaluated using the score proposed by Bond et al. (45) that allowed classifying methodologies into three categories: high (score 5–7), medium (3–4) or low (0–2) quality (see Table 1 and detailed quality score in Supplementary Material).

**Table 1:** Parameter details in original studies.

**Name**	**N (ASD/control)**	**Gender (% males)**	**Age (mean ASD/control)**	**Diagnostic method**	**Cognitive level**	**Stimuli**	**Measure**	**Skills assessed**	**Scales**	**Quality score**	**Results**
Bangerter et al. (25)	124/41	75/65.85%	Children > 6 years old and adults (14.97 ± 8.19/16.27 ± 13.18)	ASD (ADOS-2)	IQ > 60 (KBIT-2) 99.25 ± 19.25	Funny videos (America's funniest home videos' library)	Automatic facial analysis software FACET (FACS)	Spontaneous expression of Joy	SRS-2 ABC ABI	High	Lower expression of joy in ASD group (*p* < 0.05). Correlation between the activation of AU12 and ABI impulsivity and hypersensitivity. Distinction between 2 subgroups: hypo-expressive (correlation with ABC social withdrawal) and hyper-expressive (correlation with ABI impulsivity)
Capriola-Hall et al. (28)	20/20	90/70%	Children 9–12 years old (10.20/10.81)	ASD (ADOS-2)	No intellectual deficit (WASI-II) 100.55/118.15	Dynamic human faces and cartoons, emotional scene with audio	Automatic facial analysis software FEET (Kinect VT-KFER)	Voluntary expression of joy, anger, fear, neutral		High	Differences in FEE accuracy (*p* = 0.008), mainly with human faces (*p* < 0.05). More errors in the ASD group for low-intensity cartoons and high-intensity human faces. Convergence between human and computer coding (*p* < 0.001)
Del Coco et al. (29)	5/5	No data	Children 4–6 years old (5.5 ± 1.3)	ASD (ADOS-2)	Development quotient between 92 and 42 (mean 70) (GMDS)	Videos from cartoons	Automatic computer analysis	Spontaneous expression of joy, fear, sadness		Low	Higher facial expression complexity in the control group both overall and when the upper and lower face are analyzed separately. More intra-group similarity than inter-group similarity. Statistics of data were not provided.
Grossard et al. (30)	36/157	75/52%	Children 6–12 years old 8.8 ±1.8/8.4 ± 1.4	ASD (ADOS and/or ADI-R)	WISC-IV 92.5 (±17.5)	Verbal request - Dynamic Avatar faces	Automatic facial analysis algorithm (random forest classifier)	Voluntary expression of joy, sadness, anger, neutral	ADI-R sub-scores	High	More ambiguous expressions in subjects with ASD requiring consideration of more facial markers. Anger confused with joy more frequently in the ASD group.
Guha et al. (31)	24/21	No data	Children 9–14 years old	ASD	No data	Dynamic human faces (Mind reading corpus)	Facial Motion Capture	Voluntary expression of joy, sadness, anger, fear, surprise, disgust		Medium	Difference between groups (*p* = 0.024), mostly for fear, disgust and sadness, especially in the eye area. Less facial symmetry, less variation in intensity.
Guha et al. (32)	20/19	90/95%	Children 9–14 years old 12.90 ± 3.19 /12.67 ± 2.34	ASD (ADOS)	HFA (Lieter-3/PPVT-4) 106.35 ± 15.38/108.74 ± 11.93	Dynamic human faces (Mind Reading corpus)	Facial Motion Capture (FACS)	Voluntary expression of joy, sadness, anger, fear, surprise, disgust		Medium	Less complexity of facial movements in the ASD group mainly from the eye area. Significant differences in joy, sadness, disgust (*p* < 0.05) mainly because of the eye area
Landowska et al. (33)	11/8	No data	Children	ASD	?	Interactions with a robot	Automatic facial analysis software Face Reader (FACS)	Spontaneous expression of joy, sadness, anger, fear, surprise, disgust		Low	Less expression of sadness (*p* = 0.002) and disgust (*p* = 0.01) in the ASD group during evaluation.
Manfredonia et al. (34)	144/41	77.8/65.9%	Children and adults 6–63 years old (14.6 ± 7.8/16.3 ± 13.18)	ASD (ADOS)	IQ > 60 (KBIT-2) 99.2 (±19.6)	Written request	Automatic facial analysis software FACET (FACS)	Voluntary expression of joy, sadness, anger, fear, surprise, disgust	ABI SRS-2	High	Difference in the use of joy, fear, surprise and disgust AUs (*p* < 0.05), but not sadness and anger AUs. Negative correlation between some FEEs and SRS and ABI social communication subscores (mainly <13 years)
Metallinou et al. (35)	21/16	No data	Children 9–14 years old	ASD	HFA	Dynamic human faces	Facial Motion Capture	Voluntary expression of joy		Medium	More asynchronous movements between the different face regions and more variability and inaccuracy at the lower face in ASD children
Owada et al. (36)	18/17	100/100%	Adults 18–55 years old (32.2 ± 7/29.6 ± 4.3)	ASD (ADI-R and ADOS)	> 80 (WAIS) 105.8 ± 10.9	Semi-structured interview (ADOS)	Automatic facial analysis software Face Reader Noldus (FACS)	Spontaneous expression of joy, sadness, anger, fear, surprise, disgust, neutral	ADOS WHOQ-OL GAF AQ STAY-A CESD	Medium	More neutrality and less joy in the AD group with less variability (*p* < 0.05). Correlation between neutrality and higher ADOS social reciprocity subscore (*p* = 0.042).
Samad et al. (37)	8/8	No data	Children and young adults 7–20 years old (13 ± 4.4/16 ± 4.1)	ASD	No data	Static faces of 3D avatars	Facial imaging sensor 3D	Spontaneous expression of joy, sadness, anger, fear, surprise, disgust		Low	Asymmetrical facial muscle activation in ASD subjects compared to control group
Trevisan et al. (38)	17/17	76/76%	Children (10.21 ± 1.78/8.97 ± 1.30)	ASD (ADI-R and ADOS)	HFA (WASI vocabulary and matrix subtests)	Emotional videos	Automatic facial analysis software FACET (FACS)	Spontaneous expression: Positive (joy), negative: (sadness, anger, fear, surprise, disgust) neutral	AQ CAM	Medium	Negative correlation between alexithymia (CAM) and negative FEEs (*p* = 0.03), positive correlation with neutrality (*p* = 0.012) but not with positive FEE. No correlation between FEE and autistic symptoms (AQ). Difference in FEE scores between ASD and controls only for neutrality (*p* = 0.024) from univariate analysis.
Wieckowski et al. (39)	20/20	90/70%	Children 9–12 years old	ASD (ADOS-2)	HFA (WASI-II) 100.55/118.15	Dynamic cartoon and human faces Photo of emotional scene without face with audio	Automatic facial analysis software FEET (Kinect)	Voluntary expression of Joy, anger, fear, neutral	NEPSY-II	High	Children with ASD expressed accurate but more atypical FEE than controls in all conditions. Positive correlation between FEE on verbal request only and FER in the ASD group (*p* = 0.01), but not the control group.
Zampella et al. (40)	20/16	95/87.5%	Children 9–16 years old (13.8 ± 1.38/14.21 ± 2.03)	ASD (HFA) (ADOS-2 et ADI-R) SCQ	(WASI-II or WISC-IV) 108.5 ± 14.15/113.94 ± 12.68	Interactions during a conversation with a caregiver or a stranger	Automatic facial analysis software OpenFace (FACS)	Spontaneous expression of joy	SRS-2 Vineland II IRI	Medium	Children with ASD (*p*- 0.02) as well as their non-familiar interlocutor (*p*- 0.002) expressed less smiles than controls. Children with ASD showed less coordination in reciprocal smiles (*p*−0.02). Positive correlation between the coordination of smiles in ASD and social skills (SRS-2, IRI, Vineland-II).
Zane et al. (41)	19/18	89/94%	Children and adolescents 12.8/12.11	ASD (ADOS-2)	HFA (Lieter-R/PPVT-4) 105–108/110–119	Dynamic human faces (Mind Reading corpus)	Facial Motion Capture (FACS)	Voluntary expression of joy, sadness, anger, fear, surprise, disgust		Medium	Quantity of facial movements was dependent on intensity but independent of expression type (unlike the control group), and more jerky and fleeting.

*ABC, Autism Behavior Checklist; ABI, Autism Behavior Interview; ADI-R, Autism Diagnosis Interview-Revised; ADOS-2, Autism diagnosis observation schedule; AQ, Autism Quotient; AU, Action Units; CAM, Children's Alexithymia Measure; CESD, Center for Epidemiologic Studies Depression Scale; FEE, Facial Emotion Expression; FACS, Facial action coding system; GAF, Global Assessment of Functioning; GMDS, Griffith Mental Development Scales; HFA, High Functioning Autism; IQ, Intelligence Quotient; IRI, Interpersonal Reactivity Index; KBIT-2, Kaufman Brief Intelligence Test 2; Lieter-R, Lieter International Performance Scale-Revised; N, number of subjects; p, p-value; %, percentage; PPVT-4, Peabody Picture Vocabulary Test 4; SCQ, Social Communication Questionnaire; SRS-2, Social Reciprocity Scale 2; STAY-A, State Trait Anxiety Inventory; Vineland II, Vineland Adaptive Behavior Scale 2; WAIS, Wechsler Adult Intelligence Scale; WASI-II, Wechsler Abbreviated Scale Intelligence 2; WHOQOL, World Health Organization Quality of Life; WISC-IV, Wechsler Intelligence Scale for Children*.

**Table 2:** Relevant reviews and meta-analyses.

**Review/meta-analysis**	**Objective**	**Number of studies about FEE in ASD included**	**Results**	**Commentaries**
Davies et al. (22)	Review and meta-analysis of spontaneous FEE assessment in non-psychotic psychiatric disorders	6 out of 39 (including 0 using new technologies)	- Alterations of FEEs in included psychiatric disorders, except for anxiety disorders (depression, eating disorders). - Studies looking at ASD partially confirm the overall decrease in spontaneous facial expression in this group.	Review on FEEs, but is not focused on ASD or new technologies
Deutsch et al. (42)	Review of FEE assessment in people with ASD and neurobiological and clinical implications	2 Using new technologies	- Addresses the neurobiological, neuroanatomical and pathophysiological mechanisms potentially involved in ASD emotion-processing abnormalities. - Production of FERs discussed in an interventional section involving the use of new technologies.	Key considerations around recognition of FEEs and visual scanning in ASD
Keating et al. (43)	Review of FEE and FER assessment in people with ASD	17 of which 1 used new technologies	- Differences in FEEs are found between typically developing people and people with ASD, with less frequent expressions which are judged to be lower in quality by evaluators without ASD. - It seems that alexithymia can contribute to these differences in emotion expressions.	Few studies using new technologies included
Trevisan et al. (19)	Meta-analysis of studies about FEE assessment in ASD	39 out of 39 (including 1 using new technologies)	Participants with ASD produce FEEs less often and for less time. Involuntary mimicry and voluntary imitations are more often inaccurate. FEEs are also considered to be lower in quality and less precise. However, emotions are not expressed less intensely, and spontaneous reaction times are not slower.	Includes all methods of FEE analysis
Vivanti et Hamilton (44)	Review of imitation abilities assessment in ASD		- Evaluation of motor imitation skills: body, manual, language, facial. - This review suggests that most people with ASD have more difficulty in imitating unfamiliar actions and without a clear goal. In addition, imitation performance appears to be less good as social demands increase.	Reviews imitation data but not just FEEs, does not address the use of new technologies

*ASD, Autism Spectrum Disorder; FEE, Facial Emotion Expression; FER, Facial Emotion Recognition*.

## Results

### Analysis of Spontaneous Facial Expressions of Emotions

A recent meta-analytical review reported that autistic individuals display spontaneous facial expressions less frequently and for a shorter duration than in non-autistic individuals. Furthermore, they are less accurate and lower in quality (19). Only 7 studies were found to focus specifically on spontaneous FEEs in ASD using new technology.

#### Quantitative Analysis of Spontaneous FEEs in a Non-social Situation

Spontaneous facial expressions in people with ASD have been evaluated by designing various experimental conditions that are considered to provoke emotion, such as watching videos with emotional content (46), free play in children (47), using auditory (48) or olfactory (49) stimuli, or during social interaction (50). Studies using the FACS method, EMG or subjective human measures reported that subjects with ASD expressed less frequent and shorter FEEs (47, 51, 52).

The study conducted by Trevisan et al. (38) aimed to analyze automatically the quantity of spontaneous facial expressions in children with ASD using the FACS-based FACET software. In response to videos with emotional content without social interaction, the FEEs of 17 children with high functioning autism (HFA) were compared to 17 controls matched in gender and intellectual functioning. Comparisons between groups with and without ASD found differences in the production of FEEs only for neutrality (*p*η^2^ = 0.15, *p* = 0.024) but not for either positive or negative expressions. ASD children expressed more “neutral faces” than their typical peers. In addition, alexithymia scores were measured using the CAM scale (Children's Alexithymia Measure) and correlation analyses were performed with FEEs scores using covariates such as age, gender, intellectual functioning and ASD severity [Autism Spectrum Quotient (AQ)]. It was found that only alexithymia scores were predictive of the variance of spontaneous FEEs scores (*p* < 0.001, *R*^2^ = 0.346). The authors suggested that the rarer spontaneous facial expressions leading to more “neutral” facial expressions in children with ASD described clinically, was more related to concomitant alexithymia than to ASD feature severity, as proposed in a previous literature review of studies without using automated facial recognition technology (53). Alexithymia may therefore be partly responsible for deficits in both FER (54) and FEEs in ASD.

This independency of spontaneous facial expression production from ASD traits was partly replicated in a second study with a larger sample of participants conducted by Bangerter et al. (25) using the same FACET software. This study focused on the expression of joy (action units 6 and 12) in response to humorous videos in a sample of adults and children with ASD. One hundred and twenty-four subjects with ASD with an intellectual quotient (IQ) > 60 and 41 subjects without ASD were included. The authors identified an “over-reactive” ASD subgroup that statistically presented more facial expressions of joy than the control group (*p* < 0.01, *r* = 0.31) and an “hypo-reactive” subgroup that statistically presented fewer facial expressions of joy than the control group (*p* < 0.01, *r* = 0.31) (*p* < 0.001, *r* = – 0.36). No differences were found between the two subgroups in terms of IQ, severity of autistic symptoms (as measured by ADOS-2) or social skills (as measured by SRS-2), but the “hyper-reactive” subgroup differed from the “hypo-reactive” subgroup by a significantly higher ABI impulsivity score (*p* < 0.05, *r* = 0.21), while the hypo-reactive group was associated with higher social withdrawal according to the results of the Aberrant Behavior Checklist ABC (*p* < 0.01, *r* = −0.3). Consequently, the authors suggested that internalizing and externalizing symptoms associated with ASD and especially emotional regulation impairments may be more involved in spontaneous FEE deficits than autistic traits, as suggested in a previous study without the use of facial recognition technology (55).

#### Quantitative Analysis of Spontaneous FEEs in Social Interactions

In social interactions, it is often reported that individuals with ASD fail to orient their facial expressions toward their partners and have less communicative intentions. It is also frequently claimed that children with ASD tend to exhibit facial expressions less frequently in a social interaction, while other reports have suggested that these facial expressions would be less socially congruent, i.e., less appropriate to the social context. Several studies using observer ratings found that compared to a control group, children with ASD were less likely to express FEEs oriented to their mothers or other adults during interactions (39, 56, 57).

A study by Owada et al. (36) tested the use of automated emotional facial recognition technology during social interactions as part of the ASD diagnosis process. The amount of basic FEEs of 18 Japanese adult males with HFA were compared to a group of 17 non-ASD adults matched for age, gender, parental socioeconomic level, and intellectual functioning. Their spontaneous facial expressions were recorded with the FACS-based Face Reader software during a semi-structured social interaction situation extracted from the ADOS employed specifically in this study. More facial expressions of neutrality (*d* = 1.02, *p* = 0.005) and less facial expressions of joy (*d* = −0.78, *p* = 0.038) were reported in the ASD group compared to the control group. There was also less variability in the quantity of these two expressions during the transition periods between tasks in the ASD group (*p* = 0.03). This study therefore suggested a reduced facial expressiveness in social interactions, reproducing the results previously reported during a non-social situation. However, the emotional inner states and clinical characteristics of individuals were not accounted for in this study.

Beside these results, spontaneous expressions during an interaction between children with ASD and a robot were also evaluated by Landowska et al. (33) with the same Face Reader software. Interactions with a robot are supposed to be less complex and alarming than human interactions, and therefore more easily accepted by children with ASD (58). The basic facial expressions of 11 children with ASD and 8 controls were monitored to assess the amount of facial expressions recognized as valid by the software according to FACS during an interaction with the robot. The results indicated that the children with ASD expressed significantly less sadness (*p* = 0.002) and disgust (*p* = 0.01) compared to children without ASD, but the quality of the methodology in this study was considered to be low and the results need replicating.

Taken together, the above findings are in favor of a greater neutrality of spontaneous facial expressions, corresponding to the clinical “amimia of the face” described in individuals with ASD in response to non-social and social environment. This spontaneous amimia seems to be partly more related to inner emotional states or the personal ability to identify these states rather than to the severity of ASD features.

#### Qualitative Analysis of Spontaneous FEEs

Studies that have evaluated the quality of spontaneous FEEs with traditional non-automated methods have found a decrease in the quality or precision of FEEs in subjects with ASD, leading to the notion that autistic and non-autistic faces may “speak a different language” when conveying emotion. In other words, a mismatch was suggested to occur between the emotion spontaneously produced by subjects with ASD and the emotion corresponding to the same situation that would be expressed by a subject without ASD. In line with this interpretation, numerous studies have reported that autistic individuals produce spontaneous expressions that are perceived as lower in quality, and rated as odd, stilted, or mechanical by non-autistic observers and experimenters (59–61). This would explain the feeling of strangeness or ambiguity reported by non-autistic individuals when interacting with an ASD peer. Although there is evidence that autistic and non-autistic individuals exhibit expressive differences, research has not yet identified what specifically is different about these facial expressions.

Several researchers have hypothesized that the final arrangement of facial features may have spatial differences between expressions produced by autistic and non-autistic individuals (e.g., one group might open their mouth further when smiling to express happiness). One can also ask whether there might be intergroup kinematic differences (e.g., when expressing happiness, one group might break into a smile more quickly or more briefly). Although autistic and non-autistic facial expressions could, in principle, differ in terms of spatial or kinematic features, to the best of our knowledge, traditional studies with human raters or EMG paradigms have not yet specifically aimed to assess the contributions of these two factors.

Our review of new technology literature found only one pilot study from Del Coco et al. published in 2017 that used a computer-based analysis of spontaneous facial expressions of joy, fear and sadness in 5 pre-school children with ASD compared to 5 age- and gender-matched controls in response to cartoon videos (29). The different action units mobilized during viewing were analyzed using a complex algorithm developed by the authors. The data were collected with a spatial analysis approach, in terms of “global mobilization” on one hand, and “hemi part of the face mobilization” on the other (i.e., separating the upper and the lower part of the face) to compare the activation of the eye and mouth areas in both autistic and non-autistic individuals. The study concluded that non-ASD children express more complexity (calculated computationally with the average entropy) in the mobilization of their facial muscles in both the eye and mouth areas, compared to children with ASD, regardless of the emotion produced.

It was reported that facial expressions of individuals with ASD during a social interaction would also be less socially congruent, i.e., appropriate to the social context. To date, however, there is no study computing FEE quality during social interactions that reported consistent, non-subjective and accurate results. To our knowledge, no research based on new technologies has focused attention on the possible spatial and kinematic expressive differences between ASD and non-ASD individuals during a social interaction.

#### Automatic Facial Mimicry Analysis

The implicit, automatic and subconscious phenomenon of facial muscle activation in reaction to the observation of an emotional face is called “facial mimicry.” This spontaneous facial behavior corresponds to an involuntary reproduction of interlocutor FEEs and is part of the global emotional contagion existing between partners during social interactions. It is supposed to facilitate both sharing and understanding of emotional signals (62). Studies of involuntary facial mimicry in ASD have mainly used electromyography. These have shown a delay (63) or a lower expression intensity (12), with undifferentiated muscle activation, when different types of emotions (anger or joy) are expressed (64, 65) or atypical and incongruent facial expression occurs in response to a presented stimulus (9, 66) in ASD individuals compared to TD peers.

According to our literature review, one pilot study, conducted by Samad et al. (37), used non-invasive 3D facial imaging technology to measure automatic facial mimicry in response to static Avatar faces presented on a computer screen. These authors proposed a new computer vision and data mining approach that was developed from curve-based geometric features of 3D facial data in order to discern the changes in facial muscle activations during mimicry. The program captured spatial information about the face that enabled detecting and quantifying subtle changes in facial expression based on the activation of different facial muscles. Eight participants with ASD and 8 controls aged 7 to 20 were included. In the group with ASD compared to the control group, a significant increase (*p* = 0.049) in the activation of the right *levator anguli oris* (lip elevator) muscle and a decreasing tendency (*p* = 0.059) in the activation of the left *levator anguli oris* were found to occur. These results should be interpreted with caution, however, because of the small size of the monitored sample, although the authors also noted an asymmetrical activation of the facial muscles of subjects with ASD that may contribute to the impression of oddity often reported during a social interaction. 3D imaging technology has the advantage of detecting facial muscle movements in a more detailed and subtle way. However, the analysis conducted in this study was on a static image of the participant and did not address dynamic aspects of FEEs.

Whereas, the phenomenon of facial mimicry is well-documented in non-ASD subjects, traditional studies without the use of new technologies that reported reduced mimicry in children and adults with ASD did not use paradigms based on “real time” social interactions or in ecological social contexts. This was due in part to a lack of the capability to measure dynamic facial expressions throughout the course of an interaction. On the other hand, automated facial analysis via new technologies provides a rapid, efficient and objective method for measuring in natural contexts the facial expressions and their coordination produced by participant partners during such interactions.

In 2020, Zampella et al. used computer-assisted automatic detection of FEEs to analyze more specifically the coordination of facial emotions during reciprocal social interaction (40). Twenty children with HFA and 16 matched controls participated in spontaneous conversations with their mothers and with non-familiar adults. Time-synchronized videos were analyzed, and the emotion of joy was recorded by the Open Face software for each of the interlocutors as well as the coordination of the child's expression of joy with that of the adult. Both child and adult partners exhibited less positive facial expressions during conversations when the younger participant had ASD compared with the same situation when the child participant did not have ASD (*p* = 0.02). Children with ASD also manifested a less effective coordination of facial expressions over the course of conversations (*p* = 0.04). The diminished coordination in ASD participants significantly predicted scores on measures of social skills, adaptive social skills, and empathy (Interpersonal Reactivity Index IRI, *p* = 0.02). The authors suggested that the weaker effective coordination and in particular the reduced smiling ability in children with ASD also affected the responses of the social partner, in particular when it was a stranger, therefore highlighting the impact of this interpersonal process in the success of social interaction. Moreover, these findings suggested that facial emotional reciprocity is linked to socio-emotional characteristics in ASD and suggests a promising novel method for the automated monitoring of facial expressions and emotional reciprocity during natural interactions.

#### Relationship Between Spontaneous FEE Production and Social Functioning in ASD

Research using traditional analytical paradigms have provided substantial evidence that spontaneous FEE capabilities are closely linked to successful social functioning (1), and have been reported as abnormal in frequency, duration and quality in individuals with ASD, whether performing social interaction tasks or not. Task-related factors (e.g., intensity, type of emotions or stimuli: statics or dynamics) and participant characteristics were reported to strongly influence facial expressions skills in both conditions: with and without social interaction (56).

Literature based on the use of new technologies has reported reduced spontaneous FEEs in tasks without social interactions and concluded that the deficits were related to clinical participant characteristics that were not linked to ASD symptoms. These studies reported a qualitative and quantitative atypicality of FEEs that can be specified and detailed in ASD, in both spatial and kinematic aspects. This atypicality of autistic FEEs is associated with a greater expression of neutrality and asynchrony in facial expressions, which was also reported to occur during social interactions with a resultant impact on the latter' success.

It is possible that these atypical facial expressions are judged more negatively by an interlocutor and hinders the course of a social interaction or even the creation of social bonds, thereby worsening social experiences. In the study of Owada et al. (36), the intensity of the expression of neutrality was positively correlated with the reciprocal social interaction sub-score of the ADOS (ρ = 0.48, *p* = 0.042). This association in turn suggested that in ASD, a reduction in facial expressivity is associated with a deficit in *reciprocity* in social interaction skills. In the same idea, Zampella et al. reported significant correlations between poorer interaction coordination of FEEs in children with ASD during interactions and social reciprocity skills assessed by the SRS-2 score (*p* = 0.02) as well as in socio-adaptive skills assessed by the Vineland-II score (*p* = 0.001) (40). These correlations were maintained after adjusting for facial expressions intensity, indicating a relationship that is not only related to an overall reduction in facial expressivity in children with ASD. Their results further suggested that facial emotional coordination is an important process with implications for broader socio-emotional functioning in individuals with ASD and particularly for the reciprocity in social functioning. Deficits in spontaneous facial expression in people with ASD could thus be a major component of their deficit in reciprocity in social communication and interaction (19).

### Analysis of Voluntary Facial Expression of Emotions

Studies of voluntary FEEs usually separate facial imitation (according to a model: photo, video, interlocutor) and the production of FEEs in response to verbal or written requests, with the latter involving the use of one's own mental representations of each emotion. Previous studies on the developmental course of imitation skills in children with ASD reported mixed evidences for imitation impairments in children (67), although a preserved production of voluntary FEEs, particularly in adults was observed, in contrast to the impairments in spontaneous facial expressions (9, 19, 63). The authors then suggested that voluntary facial expression imitation skills may improve with age in ASD, due to social experiences. According to our review of studies using new technologies, eight studies of voluntary FEE production have been identified.

#### Voluntary Facial Imitation

Facial imitation is the ability to reproduce the facial expression presented by another in a model. In most traditional studies including adults and children with ASD, FEEs were described as less “natural” or more “mechanical” or “weird” than the FEEs of individuals without ASD, although FEEs of autistics' were still recognizable by observers (15, 59, 68). ASD individuals had more difficulty in reproducing FEEs if the stimulus was dynamic (67), and no deficit was reported when ASD individuals were asked to imitate pictures of static emotional faces (9). The quality of the FEEs were also reported to be better in adults than in children with ASD (69).

Capriola-Hall et al. (28) using automated methods analyzed voluntary FEEs production in children with ASD in response to several types of dynamic stimuli. The FEET (Facial Emotion Expression Training) software used aimed at analyzing facial information in 3D using the Kinect system and automated facial emotion recognition was conducted in real-time to provide immediate corrective feedback. Three different types of stimuli were used, involving 3 “levels”: videos of “cartoon faces” (level 1), videos of “human faces” (level 2) and finally, scenes without face, but eliciting emotion in participants (level 3). For each type of stimulus, the participant was instructed to imitate “With your face, show what I am feeling,” to produce the expected emotion. The authors hypothesized that, depending on the different type of stimulus, children with ASD produce less accurate FEEs compared to their TD peers. An overall difference in the production of FEEs was found between both groups with and without ASD (*p* = 0.008). Children with ASD produced facial emotions more often classified as “error” than the control group. This difference (*p* < 0.05) was mainly observed when the stimuli were human faces, implying that people with ASD had more difficulties in reproducing dynamic real human faces, which are more complex than cartoon representations. However, the quality of the FEEs was not assessed in this study and none of the clinical characteristics were considered to adjust the results.

The same software and method (FEET) were used in a higher quality study conducted by Wiekowski et al. (39) among 20 children with HFA compared with 20 controls aged from 9 to 12 and included a procedure with human observers/raters to determine “proof of concept” of this interactive computer-assisted program. This study's aim was to analyze both facial emotion recognition (FER) scores (assessed with the NEPSY-II Facial Affect Recognition Test) and voluntary FEE productions, then to compare the two skills in ASD. The authors reported “atypical”- but “recognizable” - FEEs, in children with ASD for both cartoon and human stimuli. When comparing automated evaluation of FEE accuracy between groups, it was found that children with ASD were as accurate as their peers when they were asked to produce a specific, named emotion (*p* = 0.54, partial η^2^ = 0.01). Children with ASD were also comparable to their TD peers when expressing emotions in response to both types of stimuli: a cartoon (*p* = 0.27, partial η^2^ = 0.03) or a human face (*p* = 0.59, partial η^2^ = 0.01). Children with ASD seemed to be less accurate at expressing an expected emotion in response to a scene without faces when the emotion was neither labeled nor modeled, although the difference was not significant (*p* = 0.09, partial η^2^ = 0.08). The authors concluded that FEEs production appeared to have deteriorated in children with ASD when facial stimuli were removed, or when the cues were less obvious. In addition, on the basis of raters' measures, the authors reported a group difference in atypicality of FEEs, since the ASD group appeared to display more atypicality in expression across all levels of the stimulus presentations (*p* < 0.01). However, even though emotion recognition scores were lower in the group of children with ASD as expected, no relationship was found between FER score and facial imitation scores.

#### Voluntary Emotional Facial Production on Request

Several conventional studies have suggested that individuals with ASD produce better voluntary FEEs when using an external model than on request, i.e., based on internal/mental representations (69, 70). Similarly, FEEs were less accurate when children with ASD were asked to display a facial expression without visual feedback, than with a mirror (71), thus providing evidence indicating that individuals with ASD rely on rule-based strategy when perceiving facial expression of emotions (72). In the absence of faces, children with ASD are no longer able to rely on such a rule-based strategy for emotional perception, resulting in a lowered accuracy (15, 59, 61, 68).

In 2019, Manfredonia et al. conducted a study including a large sample of both children and adults with HFA to evaluate their ability to produce FEEs specifically in response to a written request that did not involve a model (34). The FACET automatic facial expression analysis software was used to measure the *quality* of basic FEEs in 144 participants with ASD compared with 41 non-ASD participants. The activation of the two most representative Action Units (AU) for each emotion was recorded. Statistical analyses found significant differences (*p* < 0.05) between groups in the use of AU 12 (which mainly involves the expression of joy), AU 5 (mainly involved in fear and surprise), and AU 9 (involved in the expression of disgust). It should be noted, however, that the software's treatment of such a small number of action units could not accurately reflect either the accuracy of the FEEs or the quality itself, although potential atypicality or abnormality in voluntary activation of global facial muscle in ASD could be revealed.

#### Quality of Voluntary FEEs in ASD: What Is Atypical?

Traditional observer-based studies have failed to identify specific differences in autistic FEEs from the norm and the characteristics of FEEs responsible of the “atypicality” perceived by observers. However, through the use of new technology for motion capture with facial markers, Metallinou et al. (35) investigated more precisely the atypicality of autistic FEEs. Specifically, this study focused on the global synchronization of facial movements (35) through a facial imitation task with dynamic human faces in 21 children with HFA and 16 non-ASD children. The stimuli used were human faces expressing basic emotions taken from the Mind Reading Corpus, a video database for facial expression recognition training. The activation of a facial region, that can be measured by the intensity of movement that a given region undergoes during a facial expression, was analyzed and compared between the two groups. The results indicated a significantly reduced synchronization of motion between the upper and lower regions of the face (*p* < 0.05), and between each left/right hemiface in individuals with ASD (mouth: *p* = 0.02, eyebrow: *p* = 0.01) compared to non-ASD children. In this study, moreover, children with ASD were found to express rougher facial and head movements and especially in the lower face regions compared to typically developing (TD) children (*p* < 0.02). Complex statistical analysis also reported that children with ASD display a wider *variability* in motion intensity across facial regions compared to their TD peers, leading to idiosyncratic facial gestures that were mostly for complex expressions, such as the manifestation of joy.

These results were partly replicated in 2015 and then in 2018 by Guha et al. using motion capture analysis with the same stimuli (video clips of the six basic emotions) in a facial imitation task (31, 32). The 2015 study included 24 children with HFA and 21 controls (31). Facial Motion Capture software analyzed the whole face and eight facial sub-regions. As expected, the ASD group exhibited less efficient imitations than the control group (*p* = 0.024), especially for sadness, fear and disgust. The results also indicated differences (*p* < 0.001) in dynamic facial motions between the two groups, arising primarily from movements of the eyes. In addition, the symmetry between the right and left regions tended to be less, although this difference was not statistically significant (*p* = 0.055). Participants with ASD also produced less variability across facial regions in terms of strength of activation, leading to the conclusion that ASD subjects appear to have a less complex underlying mechanism generating facial expressions than their TD peers. This lack of complexity could at least partially explain the idiosyncratic facial motions in ASD, as perceived by TD observers. The overall differences were found to be more pronounced for negatively valenced emotions (fear, disgust, sadness), which are more likely to induce a higher perception of atypicality by observers.

In 2018 (32), the same authors questioned the clinical characteristics of their sample (20 Children with ASD and 19 TD children) and so to ensure a better group homogeneity they verified that each of the children included did not have cognitive and language disabilities, nor neurological, psychiatric or genetic co-morbidities. Differences between groups remained significant for the expressions of joy, sadness and disgust (*p* < 0.05), again observed specifically in the eye region, which exhibited most variability and complexity in the control group. The authors interpreted these findings in the context of the “eye avoidance” symptom that has been widely reported as an ASD clinical feature. They hypothesized that children with ASD may be unable to produce complex movements in the eye region, partly because of the lack of experience in perceiving and processing complex dynamic information from this region.

In 2019, Zane et al. also used Facial Motion Capture technology and the same stimuli to describe features concerning the intensity of facial expressions of emotions in individuals with ASD (41). Global facial muscle activations were recorded and subsequently analyzed using statistical growth curve analysis, in two groups of 19 children and adolescents with HFA and their TD peers, matched for age, gender, and verbal and non-verbal IQ. The authors tested whether the expression sub-type or clinical group could predict the patterns of muscle activation recorded. However, significant data were not found, and analysis failed to establish inter-group differences in terms of intensity of the FEEs produced. Contrary to previous findings, a higher variability in global muscle activations was reported in children with ASD compared to their non-ASD peers (*p* < 0.0001). Furthermore, in direct contrast to the control TD group where the pattern of facial muscle movement was positively correlated with the valence of the imitated emotion, valence provided no indication of the activation pattern of the facial muscles in the ASD group.

Significantly also, a detailed analysis of global facial motion shape showed exaggerated peaks or short apexes in the motion pattern of the ASD group, suggesting the occurrence of extreme changes in facial configuration, marked by frequent, brief spurts of large displacement, which were not observed in TD facial expressions. The authors concluded that individuals with ASD had “rougher” FEEs than their peers without ASD. Short periods of large amplitude motion may then be one of the characteristics of idiosyncratic autistic facial expressions and could contribute to negative observer judgments of facial expression quality in ASD.

From the findings of Metallinou, Guha and Zane, it could be concluded that the patterns of facial activation by individuals with ASD are more abrupt and desynchronized, involving more peaks of motion while those of the control group are more subtle and variable, implying greater complexity. It should be noted, however, that a major limitation of motion capture technology relates to the use of facial markers that can inhibit or interact with voluntary facial expressions.

Grossard et al. (30) proposed a non-invasive method for characterizing facial movements during FEE production in a large sample of 37 children with ASD and 157 typically developing children aged from 6 to 12 year old, when performing various tasks (e.g., imitation of a dynamic Avatar face and production on verbal request), while controlling several potentially confusing variables. The latter included age, sex, intellectual functioning, cultural background and the emotion subtypes (joy, sadness, anger or neutral). The automated emotion recognition processing involved the tracking and pre-processing of 49 facial landmarks that encoded geometric deformations of the face. The facial landmarks selected corresponded to strategic points such as the mouth, corners of the eyes and nose tip. The automated tracking of these landmarks and analysis (measurement of distances between markers and their orientation) generated a set of features that were distinguishable from geometric (spatial distances between markers) and appearance ones (gradients of marker orientation). A learning machine approach using Random forest (RF) classifiers was then applied.

When training and testing were performed on ASD children, the model proposal led to a poorer performance than in TD children. The FEEs produced by children with ASD were reported to be more ambiguous than those of TD children, and according to the algorithm, TD and ASD individuals did not perform similarly, except for the expression of joy. For the latter, the RF classifier needed more facial landmarks to achieve the best classification in children with ASD compared to TD children, in particular for the mouth area. This requirement was related to a hypo-activation and a higher degree of variance in motion production recorded in the eye area of children with ASD. Consequently, the algorithm essentially used the mouth to identify joy in this group, which was then categorized along with the control group, although the apparent autistic features involved less activations of the eye area muscles and a greater asymmetry. Resolving this issue therefore requires more information (via additional markers) to ensure unambiguous feature extraction and classification.

In addition, the algorithm had further difficulties in distinguishing between positive and negative expressions and confused anger and joy, classifying anger as joy more frequently in children with ASD, indicating that they had difficulties in producing FEEs with clear emotional cues, leading to ambiguity. The authors highlighted the lack of a significant effect of age or gender, but there was a significant effect of IQ on the quality of children FEE productions. Children with ASD and a lower IQ produced less accurate FEEs than those with a normal IQ, in accordance with previous findings (19).

#### Consequences for Social Functioning

Negative correlations were found by Wieckowski et al. between face activation during the voluntary production of FEEs (number and type of AUs) and socio-communicative impairments, as assessed by social communication sub-scores of SRS-2 and ABI, after adjustment for age and IQ, which was especially relevant in the case of the youngest sample participants (<13 years old) (39). The authors advocated for early specific interventions targeting an improvement of the voluntary production of FEEs in young individuals with ASD, which could potentially impact their subsequent development of social and communication skills. However, caution should be taken when interpreting these findings, that have not been replicated from now and because null results may get unreported by other authors, the link between altered FEE and social deficit in ASD should be studied more specifically.

## Discussion

### The Relevance of New Technology to Research on FEEs in ASD

The idea that people with ASD spontaneously express less frequent FEEs and of lower quality than their TD peers is widely accepted, and many screening and diagnostics tools for ASD include the items: “range of facial expression” or “inappropriate facial expressions,” as one of the early signs of ASD (e.g., the Social Communication Questionnaire, Modified Checklist for Autism in Toddlers, Childhood Autism Rating Scale, Autism Diagnostic Interview). In the line of Owada et al. (36) protocol, who tested the use of automated emotional facial recognition technology during social interactions extracted from the ADOS protocol, automated assessment of FEE algorithms could improve the (early) detection of ASD and be part of standardized diagnosis protocols for children (during social interactions between young children and clinicians extracted from the ADOS protocol for Children and toddlers, or during free interaction sessions between young children with alert signs of ASD, for example). This more automatic assessment is not supposed to confirm diagnosis but could be an interesting tool to integrate into the protocols for a more standardized and quantitative diagnosis process. It is specifically interesting in young children presenting in clinical center before the age of 5 years with various symptoms of delay or deviance in general development, communication and language, social interaction, attention and activity level, usually signaling an underlying neurodevelopmental disorder. New technology could participate to the effort of differentiate ASD, ADHD or complex and mixed NDDs, as facial expressions impairments (in contrary to facial recognition) seems to be unaffected in ADHD (73). Furthermore, it is important to promote future studies to assess the specific value of automated FEE analysis and to directly compare the accuracy of using manual human coding and computer-assisted analysis in early stage of screening process, in infants with high risk of ASD (siblings), as identification of potential prodromal symptoms of ASD.

However, research using new technologies aiming to more specifically characterize atypical autistic FEEs have reported quantitative and qualitative differences. Such approaches have found that individuals with ASD spontaneously manifest more neutral expressions (36, 38) and less expressions of joy (25, 36) than non-ASD subjects. This is consistent with the results from traditional FEEs studies, with the idea that people with ASD display less facial expressions than their peers, described as “amimia” in clinical reports (19). However, studies using new technology have highlighted factors that potentially influence spontaneous FEE productions in ASD individuals, specifically including symptoms of alexithymia (38), depression (22), and impulsivity (40), in addition to traditional factors such as age and IQ that have been usually reported in earlier studies (19). Alexithymia is characterized by difficulties in identifying, expressing, and naming emotions. Recent evidence suggests that, among both autistic and non-autistic populations, alexithymia may be implicated in a decrease in production of facial expressions. Approximately 50% of the autistic population experiences co-occurring alexithymia (56) compared to 14% of the non-ASD population (56), which is in line with the “alexithymia hypothesis” that postulates that most difficulties in emotion processing in ASD are caused by co-occurring alexithymia, rather than ASD itself (53). These results are also consistent with findings reported in populations without ASD (74).

Moreover, the literature based on the use of new technologies suggests that although a reduction in spontaneous FEE seems to be related to non-ASD clinical characteristics, a qualitative atypicality of FEEs can be identified and detailed in ASD concerning both spatial and kinematic aspects. More precisely, spontaneous FEEs in autistic individuals were found to be less complex (29), more asymmetrical (37) and less coordinated with interlocutor expressions (40). These qualitative characteristics are in part responsible for the judgment of ambiguity or oddity frequently reported by non-ASD observers and seems to worsen specifically the reciprocity component of social interaction, although these findings were obtained from relatively small samples. Future research is therefore needed to more fully characterize the spatial and kinematic expressive differences between ASD and non-ASD individuals in order to better train clinicians to “read” autistic facial expressions and to facilitate more successful social interactions early in development.

Data in the traditional literature reported that voluntary FEEs are less congruent with expected emotions or considered as less natural and odder (19) in ASD. Using computer-based emotion recognition approaches, more recent studies have objectively demonstrated FEE atypicality during various tasks such as facial imitation (28, 30, 39) or facial production without a model (34) in children with ASD. Thus far, however, research on FEE production in ASD has been almost exclusively being oriented to group comparisons between individuals with and without ASD, but the mechanisms and characteristics of FEEs that contributing to the observer's judgment of abnormality, atypicality or ambiguity have received less attention. Facial Motion Capture technologies have led to advances in the description of spatial and kinematic characteristics of atypical FEEs, revealing an asynchrony (31, 35) and a decreased degree of complexity of facial muscle activations in individuals with ASD (35, 41), particularly significant in the eye area (31, 32). This is consistent with the “eye contact avoidance hypothesis,” whereby children with ASD make rarer eye contact compared to TD peers, and therefore experience limited access to the “good model” of FEEs. The specific role of the eye region in the screening strategy on human faces has been mainly documented in TD individuals and concerns all domains of face recognition (e.g., emotions, gender or familiarity recognition) as well as its strong implication in ASD recognition impairments (75, 76). In light of these recent results, therefore, the eye avoidance hypothesis has provided a plausible explanation, not only for face recognition deficits (77, 78) but also for facial emotional production abnormalities in ASD.

Recent findings have also highlighted the role of specific action units such as AU 12, AU 5, and AU 9 in the judgment of ambiguity or the specificity of the emotion of joy, which is responsible for a high level of variability in the corresponding production of FEEs in children with ASD (34). New approaches based on the global and local analysis of facial motion have reported exaggerated peaks or short apexes in patterns of facial activation that lead to more abrupt and desynchronized expressions. These extreme changes in facial configuration, marked by frequent, brief spurts over large distances and mostly observed in the eye area, seem to be closely involved in the idiosyncrasy of autistic FEEs. These new results were replicated by Grossard et al. (30) with a mixed approach that included human raters and an innovative computerized method based on machine learning. The findings supported the hypothesis that people with ASD do not share a specific way to produce FEEs, but produce numerous variable patterns that account for the difficulty of non-ASD observers to recognize autistic facial expressions (15).

This variability in facial motion in ASD is consistent with a body of current literature that indicates a high heterogeneity in the general movement and motor profiles of individuals with ASD (79), suggesting that they move quite differently from both non-autistic individuals and other individuals with ASD. An interpretation of movement (either facial or global) can only be formulated when our interaction partner is someone who usually moves in a similar way to ourselves. Thus, movement interpretation and the resulting social interaction in autistic-autistic peers may not be any better than between autistic and non-ASD peers. This suggests that the “social deficits” in individuals with ASD could reflect, or be a consequence of, a *mismatch* in movement profiles between ASD and non-ASD (or other ASD) individuals (43). The “non-consistency” in facial motion of individuals with ASD therefore prevents the interlocutor from inferring emotional states and intentions on the basis of FEEs during social interactions.

In line with these findings, some authors have argued that impairments in voluntary FEEs in ASD indicates a degree of dysfunction in the sensorimotor processing that allows the coupling between perception and action. This coupling underlies embodied representations of emotions, the mechanistic basis of empathy in humans that relies on the human mirror neuron system (MNS) (10, 62). The human MNS is defined as a network of brain regions which are active both when participants perform an action and when they observe another person performing the same action (7). From numerous fMRI studies using emotional stimuli, the dominant theory of MNS function is based on a direct-matching model in which observed actions are directly mapped onto the observers own motor system (80). This broader mirror neuron network incorporates the somatosensory and premotor cortex and possibly the anterior insula [for a review see Hamilton (10)]. A dysfunction of the MNS may be a causal factor in poor social cognition in ASD and is commonly called the broken mirror theory (BMT) of autism. The simulation version of the broken mirror theory derives from the idea that the MNS provides the basis for simulating other people. Such simulation could apply to actions, but also to emotions and mental states. The second version of the BMT, called the chaining version, is a more subtle hypothesis based solely on the idea that some mirror neurons represent action chains or sequences, and suggests that the comprehension and production of action and emotional expression sequences is abnormal in ASD (81). Automated assessment of voluntary FEEs in ASD has provided new evidence for abnormality in the sequence of muscle activation (spatial and kinematic chain motion dysfunctions) (31, 32, 35) and for an impact on interlocutor behavior during social interaction. This is in line with both versions of the BMT in ASD, even though over two decades of research the broken mirror theory of autism is still debated.

Until now, FMRI studies that have examined mirroring responses to emotional stimuli in ASD on the basis of emotional facial viewing tasks, have provided a mixed picture, with some results suggesting a clear deficit (82), whereas others have shown normal responses under neutral or instructed task conditions (83). Group differences emerged in brain regions associated with theory of mind, but not in the inferior frontal cortex, the core component of the human MNS. In particular, both autistic and non-autistic participants engaged the inferior frontal gyrus (IFG) of the left hemisphere when instructed to attend to their own emotions, and the authors concluded that participants with ASD can engage their MNS when the task requires latter. The chaining broken mirror theory claims that these simple actions do not require chaining, whereas the production and comprehension of more complex action sequences, such as facial emotion, does require chaining which would be abnormal in ASD. Evidence relevant to the chaining theory can also be found in studies of motor control and action understanding. Individuals with autism often present comorbid dyspraxia or other motor control difficulties (84, 85), and these could be accounted for by difficulties in action chaining. Together, these findings from cognitive neuroscience and psychology suggest that perceptual systems for facial expression recognition are linked with motor as well as somatosensory feedback systems, with motor production playing an important role in the development of perceptual recognition abilities (86, 87).

Some hypothesized that atypical FEEs could also be attributable to global neuromuscular dysfunctions (i.e., not just facial muscle coordination problem) as evidences of muscles tones abnormalities in ASD were reported in the literature (88), or directly linked to face recognition deficit and/or global Biological Motion (BM) perception impairment in ASD, in line with the Mirror Neuron System (MNS) impairment theory in ASD. This hypothesis is in accordance to various studies that reported reduced ability in extracting social information from global Biological Motion, in ASD (89, 90). More recent results of a meta-analysis on global motion perception in ASD reported as well overall differences between ASD and non-ASD individuals in global motion processing with a general decreased performance in detecting or recognizing global motion patterns in perception paradigms including social but also non-social stimuli (91). Future studies, in line with Yeung et al. (92) should focus on relation between FEE, Facial Emotion Recognition (FER), global facial perception (part of BM) and global motion perception.

### New Technology for Clinical Practice

Advances in automated facial expression recognition technology should open new avenues toward clinical interventions that target these deficits. The development of such interventions based on facial expression production would be further enhanced by extending expression recognition technology to provide real-time feedback from to a subject's own facial expressions. On the basis of recent findings using this new technology, evidence suggests that practice with expression production may not only influence production itself, but may also influence perception due to possible associations between recognition and motor production, as illustrated by rare previous results obtained in non-ASD children (93, 94). Only a few studies have focused on the relationship between learning in facial motor production and learning in facial expression perception, challenging the hypothesis that motor production contributes to perceptual improvement. Further work is now necessary to test the perception-action learning and action chaining theories. Advances in these areas will lead to a better understanding of what is really different about action systems in autism, which in turn should lead to more productive interventions that help individuals with autism to imitate and interact with the social world.

Overall, this review highlights the finding that automated facial expression analysis has an efficiency equivalent to human observation rating or EMG measurements (28, 30) while being more objective, precise and less consuming in time, material, and human constraints. This has direct implications in clinical practice through this technology's potential participation with FEE computer-based analysis in the ASD diagnosis process, in line with Owada's approach (36).

ASD is an heterogeneous condition, with significant risks of added co-morbidities (95) that in call for individualization both in the initial evaluation process and in terms of socio-emotional supports. Additionally, a voluntary adaptation of facial expressions is reported to be frequently used in daily life by individuals with ASD, mainly in subjects without intellectual deficits and females (96). Self-training of their own voluntary FEEs is reported to be a strategy for compensating (“social camouflage”) for associated socio-communicational deficits. However, this requires significant effort, which could be at the origin of increased stress levels and higher levels of anxiety in subjects with ASD (97).

From our literature review, it is evident that machine learning technology may provide promising advances to individualized care (30), and offer a promising tool to support the learning process related to emotion production and correction. The inclusion of individualized computer feedback could be part of a global support strategy that could be proposed as early as possible to children with ASD, in order to diminish stress and emotional co-morbidity in later adulthood (95).

In addition, until recently, studies on the field seemed “to ignore the fact that social interactions are exactly that, an interaction between individuals,” as reported by Keating, in his recent review (43). This review focused on the “bidirectional” approach to evaluate social interactions that leads to a consideration of both sides of the interaction and discussed the small amount of research investigating non-ASD individuals' recognition of autistic expressions of emotion. Evidences that non-ASD individuals shown difficulties in recognizing ASD facial expressions are recent and rare (15, 59), but as proposed by some authors (43, 98) as social interaction is bi-directional, non-ADS individuals' recognition of autistic expressions may contribute to social interaction deficit and could be a target for future interventions. 3D sensor cameras such as Microsoft Kinect™ and the newer Intel RealSense™ received recently, much attention due to its advantages in capturing fine-grained skeleton and facial landmark data in contexts closer to real-life, providing motion facial data for more accurate analysis (35) than human observers (98). Some new recent findings and a pilot study proposed the integration of a naturalistic multi-sensory environment through the use of portable technologies including automatic facial expression recognition to help non-ASD individuals to read the emotions of children with ASD (99). Authors focused on training individuals without ASD to recognize the expressions posed by 6 children with ASD, with the use of a Kinect camera and the programmable and portable RealSense camera when viewing cartoons. More research, which formally uses a control group in their statistical comparisons, is necessary to develop this interesting approach.

### Limitations of the Review

Even though there is consensual interest in the use of new technologies for research on individuals with ASD, this review has highlighted the current limited number of studies, which have mostly been pilot studies or with small sample sizes. Moreover, the methodologies and tools employed in these studies were highly heterogeneous, with results rarely replicated and the quality relatively low (low or medium scores for 66% of the studies), making the conclusions difficult to generalize. It is therefore necessary to replicate these results with larger samples, including matched control groups (in terms of age, IQ, gender and alexithymia). However, the increasing number of studies, mostly published in the last 2 years, suggests that new valid proposals and data will emerge in the near future.

In addition, future studies need to consider participant characteristics factors. First, intellectual functioning has not been consistently assessed, with studies to date having mostly included individuals with high functioning autism only. Accordingly, the majority of the findings reviewed may not be generalizable to individuals with ID. Secondly, as widely reported and relative to the neurodevelopmental aspect of ASD, age is a factor that strongly influences the ability to produce FEE (19). Studies have often chosen to include only children or adults, but it would be instructive to conduct longitudinal studies to better address the developmental course of FEE skills and capability. Furthermore, gender differences in emotional expression have been reported in typically developing children (100) and the effect of gender on autistic symptomatology is an issue raised by many authors (101), and is therefore a factor which also needs to be considered in studies of FEEs in individuals with ASD. Moreover, only a few studies considered symptoms of depression, alexithymia, level of empathy and emotional regulation abilities, despite an impact of these participant characteristics on FEEs (22, 25, 38, 40). Thus, the identification of subgroups of individuals with ASD, as proposed in recent studies using automatic FEE detection (25, 41), may help to explain some of the conflicting findings in the traditional literature. The inherent heterogeneity of ASD and expression of autistic symptoms, including the idiosyncratic production of FEEs, must be taken into account to better interpret the results of clinical research.

Moreover, most studies in the field of new technologies have focused on the technological development of software rather than on their clinical application, and have tended to be reported in journals specializing in computer science or engineering, which perhaps does not promote accessibility to the wider experimental research and clinical community. Although several recent studies have found that individuals with ASD display a good acceptance and interest in technology-based interventions (102), there is still little proof of concept, and only a few researchers have tested their approaches and the efficacy of computer-based FEE training on social and perceptual clinical functions. Translational approaches must also be further developed to convince clinicians, and promising tools should be tested using methodologies that are accepted by both clinicians and researchers alike, so that they can be more widely applied.

### Limitations in the Use of New Technology

The use of new technologies to analyze the production of FEEs in people with ASD is a promising avenue, both in terms of gaining fundamental neurobiological knowledge of emotion processing and also in the study of learning and plasticity in perception and production systems. Such innovative technology could contribute not only to potential new therapeutic approaches to support social impairments in ASD, but also to train and support caregivers and clinicians to “read the language” of autistic facial expressions that might be lead to a reduction in bidirectional socio-communicative difficulties.

Nevertheless, the ethical question of computer and robotic usage arises in the field of health care, particularly regarding automated facial recognition. The protection of private data represents a challenge in the health field and more particularly when data requiring subject identification is involved, as with facial recognition measures (103). Consequently, the employment of these approaches requires special attention to be paid to ensuring patient information confidentiality and consent, as well as the implementation of rigorous procedures to ensure the security of computerized health data storage.

## Conclusion

Most research focusing on facial emotion processing in ASD has emphasized facial emotion recognition differences, and only a few studies have addressed features of the actual production of FEEs in individuals with ASD, despite its major role in social functioning (1, 12). Most traditional studies conducted over the past few decades have agreed that ASD individuals exhibit more atypical or less frequent spontaneous and voluntary FEEs (19). Recent studies using new technologies have proposed advances in the categorization and description of “what is abnormal” in autistic FEEs, through spatial and kinematic computer-based analytical approaches. Current results indicate (i) that participant characteristics are strongly implicated in spontaneous FEE production, as age, IQ, but also symptoms of alexithymia depression, and impulsivity (ii) that asynchrony and deficits in eye region activation could be specifically involved in the abnormal production of voluntary FEEs and feelings of “strange faces” in non-ASD individuals, contributing to deficit in social reciprocity, (iii) that atypical FEEs may be attributable or linked to global Face Recognition deficit and/or global Biological Motion (BM) perception impairment in ASD as well as sensorimotor processing impairments in line with the Mirror Neuron System (MNS) impairment theory in ASD. Some new recent findings based on portable technologies that permit the integration of a naturalistic multi-sensory environment recording, including automatic facial expression recognition, are promising tool to address the issue of FEEs impairments in ASD, in a global, bidirectional approach that leads to a consideration of both sides of the interaction to explore also how non-ASD individuals may be trained to the ASD atypical affective expressions recognition.

## Data Availability Statement

The original contributions presented in the study are included in the article/Supplementary Material, further inquiries can be directed to the corresponding author/s.

## Author Contributions

KB and AA: conception of the review, analysis and interpretation of data for the work. AP and MB: contributed to manuscript revision and provided approval for publication of the content. All authors contributed to manuscript revision, read, and approved the submitted version.

## Conflict of Interest

The authors declare that the research was conducted in the absence of any commercial or financial relationships that could be construed as a potential conflict of interest.
