# Supplementary material for: New Technologies as Promising Tools for Assessing Facial Emotion Expressions Impairments in ASD: A Systematic Review
Source: Front Psychiatry. 2021 May 5;12:634756. doi: 10.3389/fpsyt.2021.634756 (PMC8131507; doi:10.3389/fpsyt.2021.634756)
Supplement: Supplementary file 1 [file Data_Sheet_1.PDF]

Supplementary material: quality score of studies included in the review (Bond et al. 2013)

	**Criterion**														Total		Quality
**Name**	Use of a randomised group design		Focus on a specific, well-defined disorder or problem		Comparison with treatment-as-usual, placebo, or less preferably, standard control		Use of manuals/ protocol/ training		Fidelity checking procedure/ supervision of intervention		Sample large enough to detect effect (from Cohen, 1992)		Use of outcome measure(s) that has demonstrably good reliability and validity<br>*(2 points if more than one measure used).*				
	R1	R2	R1	R2	R1	R2	R1	R2	R1	R2	R1	R2	R1	R2	R1	R2	
Bangerter et al. [2020]	0	0	1	1	1	1	0	0	1	1	1	1	1	1	5	5	High
Capriola-Hall et al. [2019]	0	0	1	1	1	1	1	1	1	1	0	0	1	1	5	5	High
Del Coco et al. [2017]	0	0	1	0	0	1	0	0	0	0	0	0	0	0	2	1	Low
Grossard et al. [2020]	0	0	1	1	1	1	1	1	1	1	1	1	0	0	5	5	High
Guha et al. [2015]	0	0	1	1	1	1	1	1	1	1	0	0	0	0	4	4	Medium
Guha et al. [2018]	0	0	1	1	1	1	1	1	1	1	0	0	0	0	4	4	Medium

Landowska et al. [2020]	0	0	1	1	1	1	0	0	0	0	0	0	0	0	2	2	Low
Manfredonia et al. [2019]	0	0	1	1	1	1	0	1	1	1	1	1	1	1	5	6	High
Metallinou et al. [2013]	0	0	1	1	1	1	0	0	1	1	0	0	0	1	3	4	Medium
Owada et al. [2018]	0	0	1	1	1	1	1	1	0	0	0	0	1	1	4	4	Medium
Samad et al. [2015]	0	0	1	1	1	1	0	0	0	0	0	0	0	0	2	2	Low
Trevisan et al. [2016]	0	0	1	1	1	1	1	1	0	0	0	0	1	1	4	4	Medium
Wieckowski et al. [2019]	0	0	1	1	1	1	1	1	1	1	0	0	1	1	5	5	High
Zampella et al. [2020]	0	0	1	1	1	1	0	0	1	1	0	0	1	1	4	4	Medium
Zane et al. [2019]	0	0	1	1	1	1	1	1	1	1	0	0	0	0	4	4	Medium

R1 = reviewer 1 (KB), R2 = reviewer 2 (AP)
